# Preliminary effect of projectile yaw on extremity gunshot wounding in a cadaveric animal model: a serendipitous study

**DOI:** 10.1007/s00414-020-02271-7

**Published:** 2020-03-11

**Authors:** Tom Stevenson, Debra J Carr, Iain E Gibb, Sarah A Stapley

**Affiliations:** 1 Impact and Armour Group, Centre for Defence Engineering, Cranfield University, Defence Academy of the United Kingdom, Shrivenham, SN6 8LA UK; 2 Cranfield Forensic Institute, Cranfield University, Defence Academy of the United Kingdom, Shrivenham, SN6 8LA UK; 3 Present Address: Defence and Security Accelerator, Porton Down, Salisbury, SP4 0JQ UK; 4 Centre for Defence Radiology, at c/o Sickbay HMS Nelson, HMNB Portsmouth, Hampshire, PO1 3HH UK; 5 Royal Centre for Defence Medicine, ICT Building, Research Park, St Vincent Drive, Birmingham, B15 2SQ UK

**Keywords:** Yaw, Gunshot, Wounding, Clothing, Extremity, AK74

## Abstract

Gunshot wounding (GSW) is capable of causing devastating tissue injuries by delivering kinetic energy (KE) through the contact surface area of a projectile. The contact surface area can be increased by yaw, deformation and fragmentation, all of which may be caused by any intermediate layers struck by the projectile prior to entering its target. This study aims to describe whether projectile yaw occurring before penetration of a cadaveric animal limb model causes greater damage with or without clothing layers present using 5.45 × 39 mm projectiles. In total, 12 fallow deer hind limbs were shot, further divided into 4 with no clothing layers (C_nil_), 4 with a single clothing layer (C_min_) and 4 with maximum clothing layers (C_max_) as worn on active duty by UK military personnel. Contrast computed tomography (CT) of limbs was used to measure permanent cavity size and the results were compared using analysis of variance (ANOVA). No significant differences were found among clothing states for each series of measurements taken, with greater cavity sizes noted in all clothing states. This is in contrast to previous work looking at symmetrically flying projectiles in the same model, where a larger permanent cavity was found only with C_max_ present. Projectile yaw is therefore likely to be a key variable with regard to causation of damage within this extremity wound model.

## Introduction

Wound ballistics study can be challenging to the modern researcher. With the variables that require control in order to preserve objectivity and scientific rigour, reproducing high-quality experiments is arduous for any researcher. With previous studies having explored or commented upon the survivorship burden from conflicts throughout the twentieth century, extremity gunshot wounding (GSW) is often noted to make up the largest proportion of injuries [1–8].

With prior research from this group having modelled extremity GSW to test the effects of UK military clothing on wounding patterns, key variables such as velocity, engagement distance and yaw have been previously controlled [9, 10]. With respect to projectile yaw, when considering military projectiles such as 7.62 × 39 mm or 5.45 × 39 mm, unopposed projectiles in flight are base-heavy and ultimately will yaw away from the central axis and lose flight stability [11]. With regard to wounding potential, the greater the contact surface area of a projectile (i.e. its shape, stability and integrity e.g. deforming or fragmenting) with its target will mean a greater amount of kinetic energy (KE) delivered over a fixed distance by a known velocity and mass of the projectile [12–19]. Under these circumstances, the property of interest is kinetic energy density (KED). This is defined as the energy at impact divided by the presented area of the projectile [20]. Open literature pertaining to the effects within a target of projectiles yawing prior to target strike is sparse. One study by Wen et al. in 2017 describes the effect of preliminary yaw from a computer model using 7.62 × 39 mm projectiles based on a gelatine model. The study observed that greater projectile yaw on striking the target leads to the projectile reaching maximum yaw (90°) over a shorter penetration depth and therefore delivering a greater KE load to the model [21]. Intermediate layers such as clothing can destabilise projectiles in flight such that they yaw sooner than if they struck a bare target [9, 10, 22]. This would also therefore lead to yaw occurring sooner within the target and thus allowing for a greater delivery of KE and subsequently greater wounding potential. Other work has looked at the effect of projectile yaw on armour penetration; for example, using 7.62 × 54R mm projectiles with small amounts of yaw induced prior to target strike was found to increase penetration of certain armour materials [23, 24].

The aim of this preliminary study was to describe whether projectile yaw occurring before penetration of a cadaveric animal limb model causes worse damage with or without clothing layers present using 5.45 × 39 mm projectiles.

## Materials and methods

Ethical approval for this work was granted through Cranfield University Research Ethics System (CURES/3579/2017).

### Materials

**Fig. 1:**
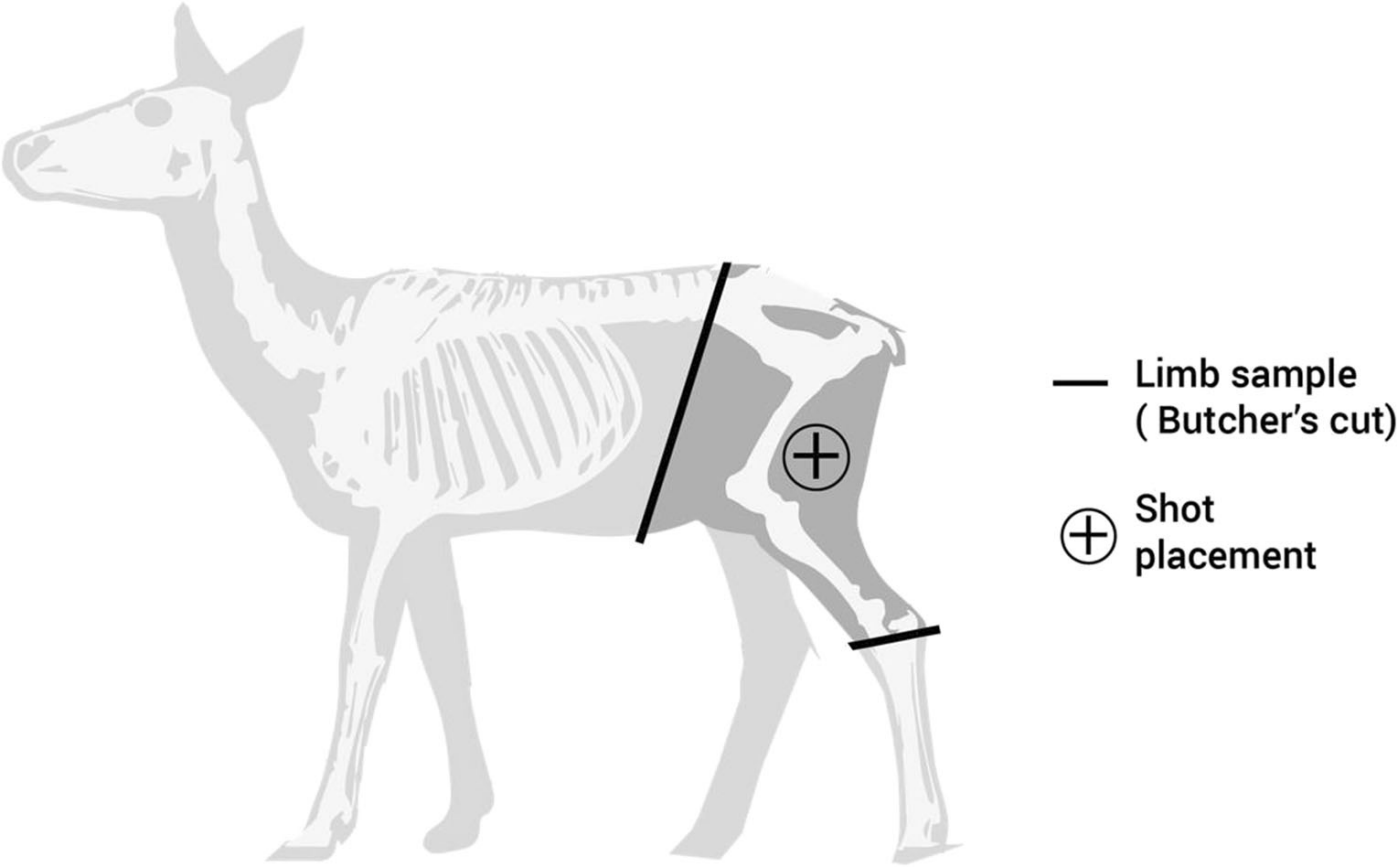
Fallow deer anatomy schematic demonstrating limb preparation and shot placement

The materials chosen for study were from previous work by this group [9, 10, 25]. Using Multi-Terrain Pattern (MTP) UK standard issue military clothing to provide the intermediate layers, the clothing was prepared in two states, the minimal state (C_min_) and the maximal state (C_max_), to be compared with a bare control (C_nil_) (see “Methods” below). Ammunition was quarantined by batch to ensure physical property differences could be kept to a minimum [26]. The ammunition type selected was a 5.45 × 39 mm mild steel core projectile, a typical threat faced during recent conflicts by UK forces [5, 27], and used in previous work by this group.^1^ Whilst there are multiple commonly used human tissue surrogates for ballistic studies, such as gelatine or soap as synthetic tissues, or porcine limbs as cadaveric and live animal tissues, there are advantages and disadvantages associated with each which are detailed within a recent comprehensive review [28]. Where gelatine is validated against live porcine thighs [14, 18], and it is known that porcine tissues have a thicker skin and subcutaneous tissues compared with human [28], the authorship of this work required the use of a human tissue surrogate more biofidelic and representative of a healthy military population. The use of deer limbs for ballistic research have been described as a comparable human tissue surrogate and validated within previous research [10, 25, 29, 30]. Therefore, the animal tissue chosen for this testing was fallow deer (*Dama dama*) hind limbs. Limbs were of a mass of 9.5–13 kg and measured approximately 280 mm × 700 mm × 100 mm (width × height × thickness). Limbs were culled for entry into the human food chain rather than specifically for research, and prepared by a professional butcher (Fig. 1). Limbs were used as both fresh targets (within 72 h of culling) and also defrosted to room temperature from freezer storage over a 72 hour period due to availability of range facilities versus limb acquisition. Differences in ballistic effects between fresh and defrosted frozen cadaveric material have previously been shown to be negligible [31].

### Methods

**Fig. 2:**
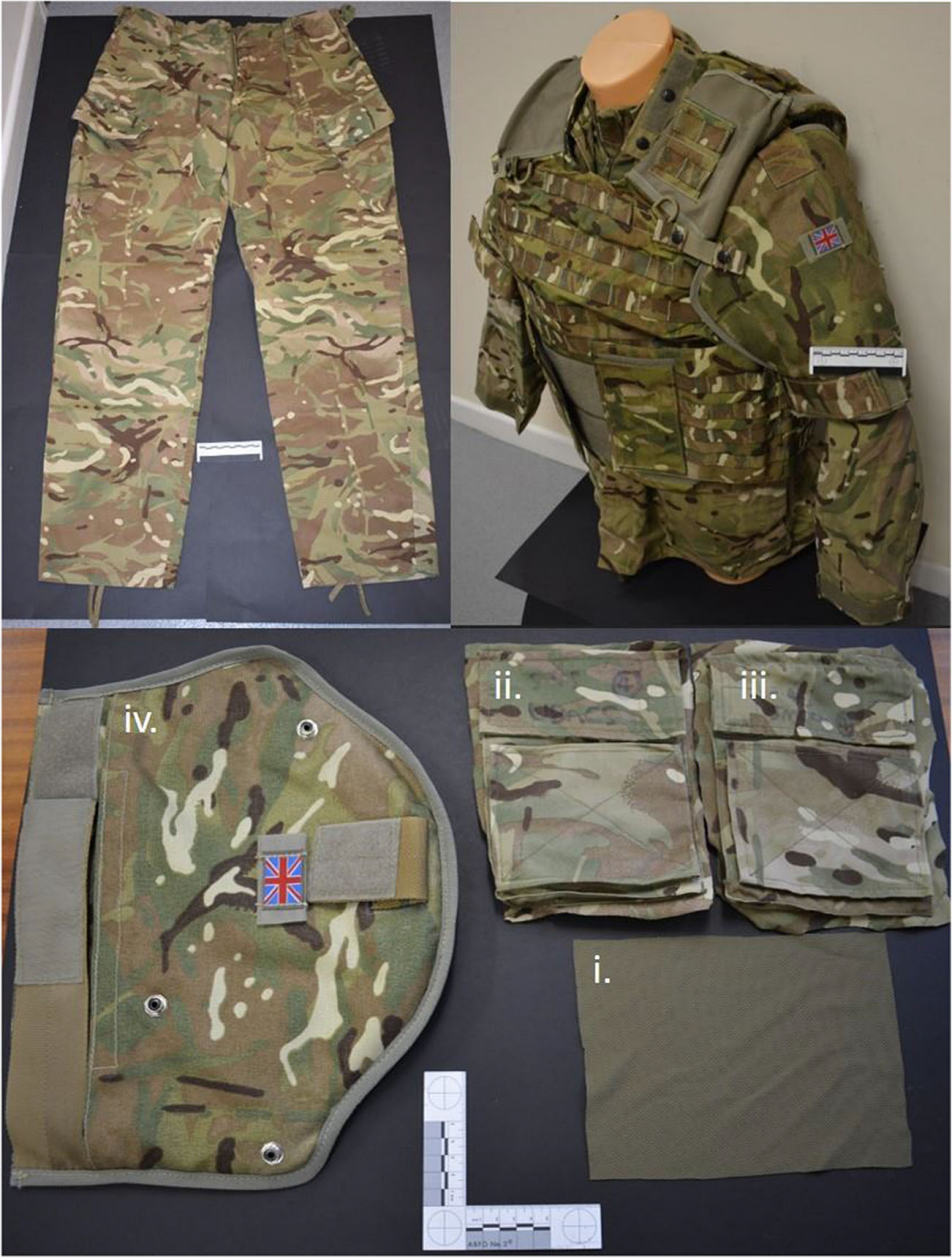
Examples of MTP clothing used. Clockwise from top left: MTP trousers; top right: t-shirt, UBACS, smock and brassard as worn by service personnel; bottom: (i) t-shirt, (ii) UBACS, (iii) smock and (iv) brassard layers prepared for testing

**Fig. 3:**
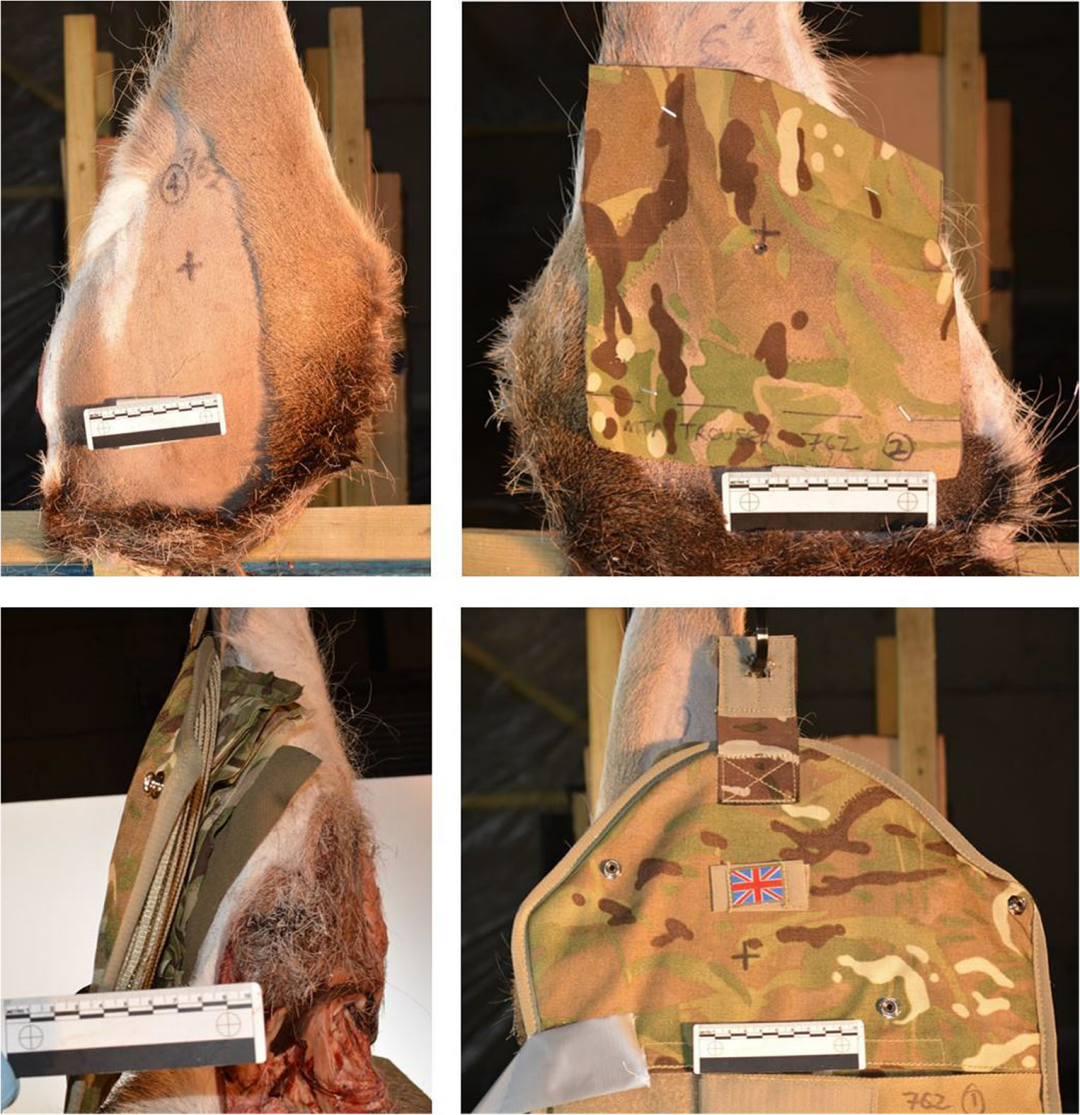
Clockwise from top left: C_nil_ front view; C_min_ front view; C_max_ front view; C_max_ side view

The method for laundering and preparing the clothing states, including fabric analysis, and preparing the limbs was as described in previous work [9, 10]. A minimal clothing state (C_min_) was required, consisting of a single layer of MTP clothing taken from issued trousers, and also a maximal clothing state (C_max_) consisting of the combined layers of clothing taken from an issued t-shirt, Under Body Armour Combat Shirt (UBACS), smock and upper arm brassard as worn on duty by UK service personnel (Fig. 2). These were then compared with bare samples with a zero clothing state (C_nil_) as a control. Fabric samples for C_min_ were cut from laundered MTP trousers (250 × 250 mm)^2^ and pinned to the front face of the relevant deer limbs (Fig. 3, top right image). Fabric samples for C_max_ were measured and cut in relation to the upper sleeve pocket size on the UBACS and Smock (200 × 150 mm),^3^ and placed in layers with the t-shirt layer innermost, then UBACS, smock and finally with the brassard then placed over the top of the other layers (Fig. 2 lower image and Fig. 3 lower images).

Four limbs were prepared for C_min_ and C_max_ clothing states, respectively, compared with four limbs with C_nil_ (i.e. bare limbs) giving a total of 12 limbs. Limbs were all shaved on the lateral surface, and suspended upside down using an “S”-shaped metal hook looped between the distal tibia and fibula at the ankle joint.

**Fig. 4:**
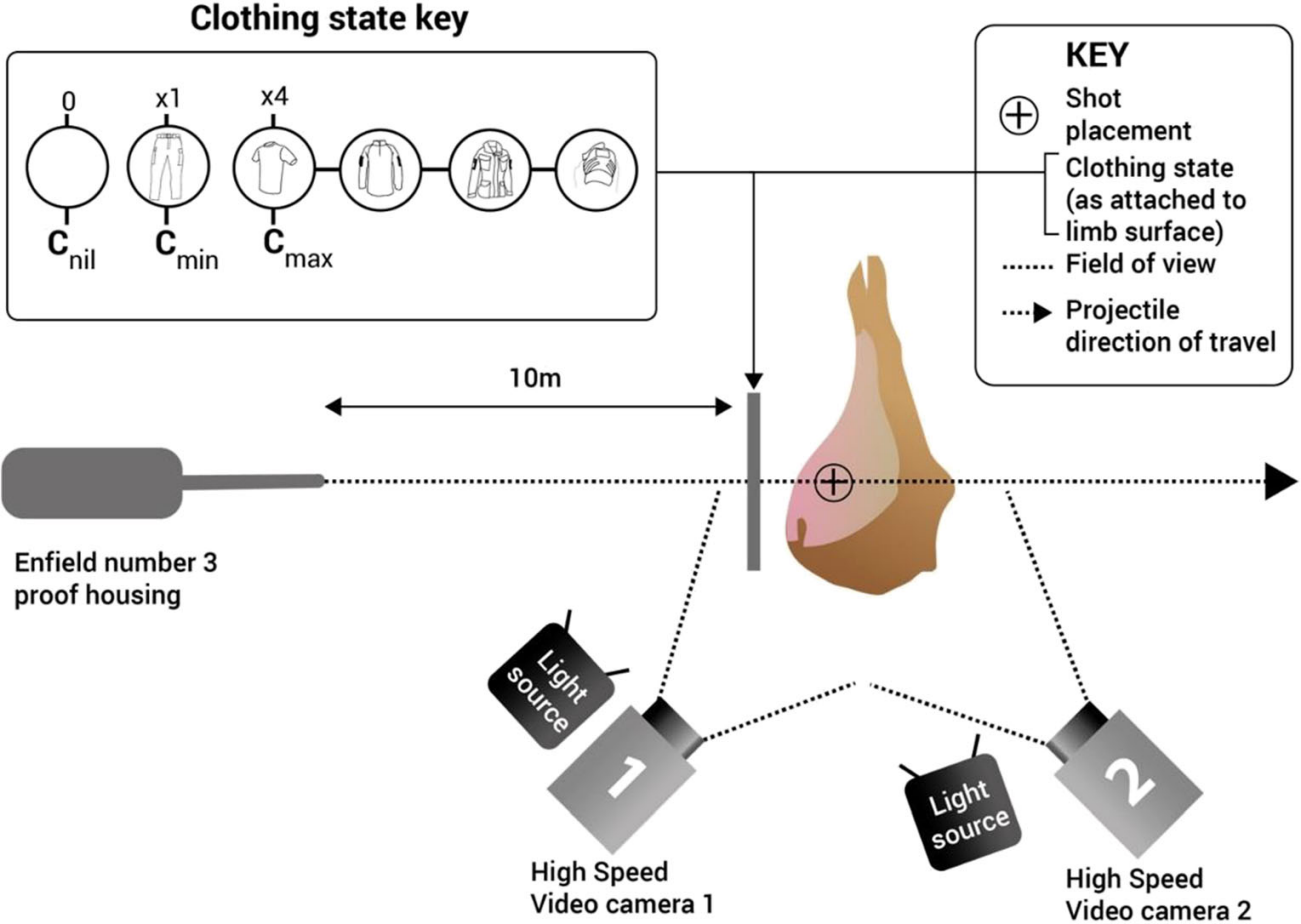
Schematic demonstrating the experimental set up

Projectiles were fired from a number 3 proof housing on an indoor range with limbs set at 10 m from the end of the barrel. Projectile yaw prior to striking the target was induced serendipitously by firing the 5.45 × 39 mm projectiles from a barrel intended to fire 5.56 × 45 mm projectiles. The resultant precession and nutation prevented flight stabilisation, and allowed projectiles to yaw by several degrees prior to striking the targets. No facility to measure yaw angle was present as it had not been a part of the initial experimental design. Each limb was perforated once by a 5.45 mm projectile, with shots aimed to strike the lateral surface of the hind limb, travelling through the soft tissue compartment posterior to the femur (Fig. 4).

Impact velocities for all projectiles were measured using Doppler radar (Weibel W700). A high-speed video (HSV) was used to capture the event in real-time, showing the external wounding patterns of the limbs from both the entrance^4^ and exit^5^ surfaces using camera 1 and camera 2, respectively (Fig. 4). GSW patterns were qualitatively examined using Phantom Software (Visions Research, Phantom Camera Control Application 2.6).

**Fig. 5:**
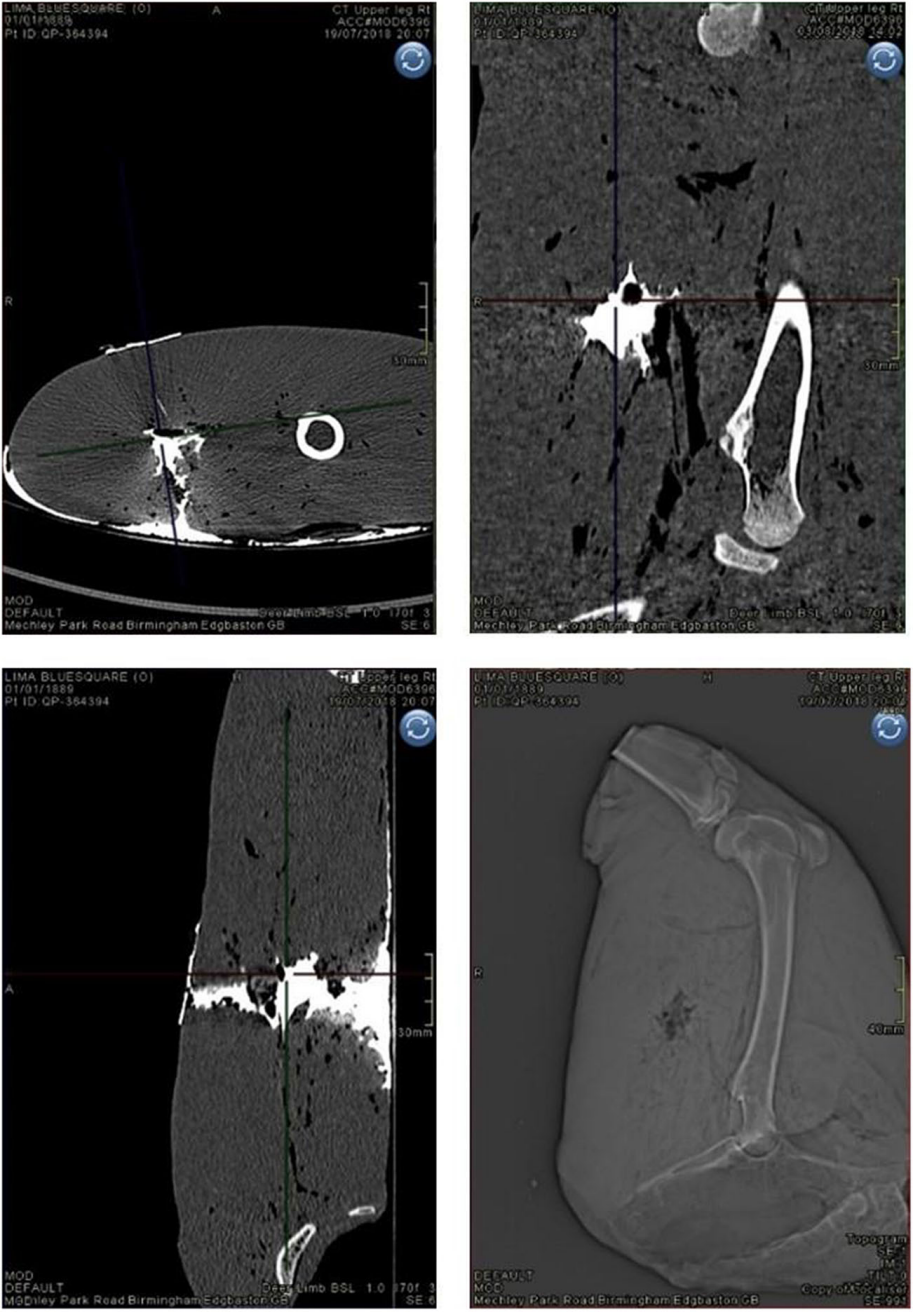
Clockwise from top left: contrast image, axial plane; contrast image, sagittal plane; CT scout view, sagittal plane (prior to contrast); contrast image, coronal plane

**Fig. 6:**
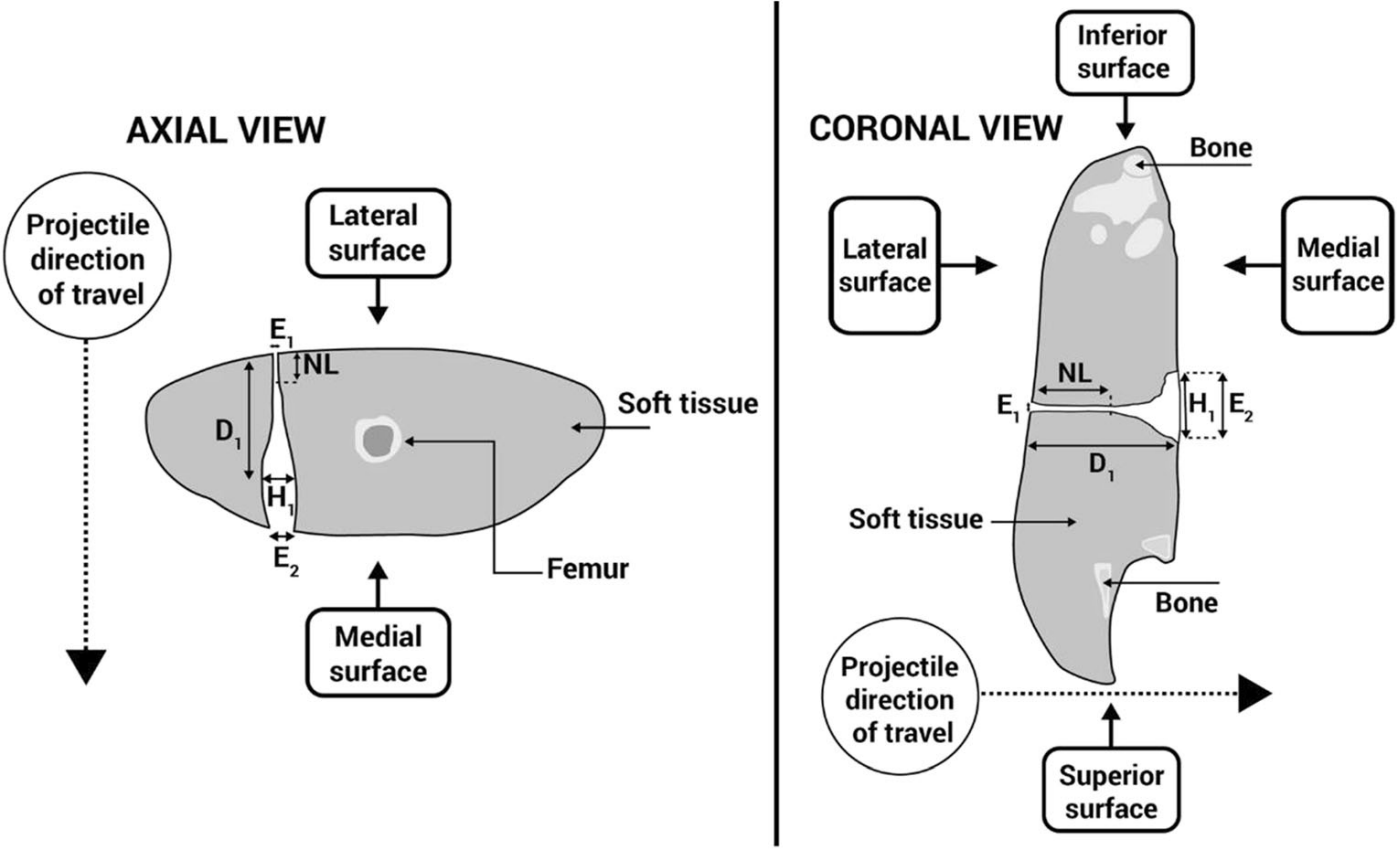
Schematic demonstrating CT scan measurements taken in axial and coronal planes of view in this example schematic, H1 and E2 in the coronal view were the same; however, this varied amongst specimens)

All limbs underwent photography post-shoot, using a Canon D5100 Digital SLR camera (S/N 6773411). Damage within limbs was measured using contrast enhanced computed tomography (CT) with a protocol developed in previous work [25]. The CT scanner used was a dual source (2 × 64 slice) Siemens SOMATOM Definition MSCT scanner (System SOMATOM Definition AS, 64622, Siemens AG, Wittelsbacherplatz, DE – 80333 Munchen, Germany). Scans with and without contrast used a standard adult pelvis protocol (exposure figures were 120 kV and 25–32 mAs) with 1.0 mm slice soft tissue and bone reconstructions in the axial, sagittal and coronal planes. Contrast injected into wounds consisted of 10–20 mls of Omnipaque 300 contrast (OMNI300, GE Healthcare) until spillage at the exit wounds was seen. The dimensions of damage measured were in both axial and coronal viewing planes using multi-planar reconstruction (MPR) images (Fig. 5) within the AGFA Enterprise Imaging Patient Archive and Communications System (PACS). The damage dimensional measurements of the GSW patterns were as follows: the neck length (NL), maximum height of the permanent cavity (H1), distance to maximum height of the permanent cavity (D1), entry wound diameter (E1) and exit wound diameter (E2) (Fig. 6).

### Statistical analysis

The International Business Machine Corporation’s Statistical Package for Social Services version 24 (IBM SPSS Statistics v24), analysis of variance (ANOVA) was used to determine the effect of the different clothing states on NL, H1, D1, E1 and E2. The damage measurements taken from axial and coronal viewing planes were considered together, as were damage measurements from the different clothing states. Homogeneity of variance and normality of data were confirmed with a significance level of 0.05 applied. Significant differences due to clothing state were identified using Tukey’s honest significant difference (HSD) test. Main effects and significant interactions only are discussed in the results section.

## Results

Mean impact velocity for the 5.45 mm projectiles was 907 m/s (SD = 6 m/s). Each limb was perforated by its respective projectile. No projectiles appeared to fragment from review of the HSV, and of those projectiles recovered from the bullet trap (*n* = 10), there did not appear to be qualitative evidence of deformation or fragmentation.

Evidence of bullet wipe and yarn pull-out on the surfaces of the fabric samples was consistent with that described within the literature [11, 32–34].

The dimensions collected for the damage to limbs caused by 5.45 × 39 mm projectiles for all clothing states are summarised in Table 1. Where an inequality of error variance in ANOVA testing for exit wound dimensions was found due to the relatively high coefficients of variation (CV) seen, areas of the exit wounds were calculated (EA) and are shown, along with raw exit wound dimensional data in Table 2. ANOVA results are given in Table 3 below; data subgroups identified by Tukey’s HSD are also included.

**Table 1:** Mean, standard deviation (SD) and coefficient of variation (CV) for dimensions measured

Projectile / clothing state	NL			D1			H1			E1			E2		
	Mean (mm)	SD (mm)	CV (%)	Mean (mm)	SD (mm)	CV (%)	Mean (mm)	SD (mm)	CV (%)	Mean (mm)	SD (mm)	CV (%)	Mean (mm)	SD (mm)	CV (%)
5.45 mm / C_nil_	44.4	22.5	50.6	69.7	19.8	28.5	17.2	3.7	21.5	5.1	0.9	18.4	18.9	3.7	19.4
5.45 mm / C_min_	31.4	31.9	101.6	68.6	22.1	32.2	16.6	4.0	24.0	6.7	3.8	56.9	15.6	5.8	37.0
5.45 mm / C_max_	18.8	21.5	114.7	62.5	26.9	43.1	22.7	8.9	39.4	7.9	4.3	53.7	23.4	9.1	39.0

**Table 2:** Exit wound dimensional measurements taken from CT scans

	Clothing state											
	C_nil_				C_min_				C_max_			
Limb number	1	2	3	4	5	6	7	8	9	10	11	12
Exit (axial view) (mm)	22.2	15.5	22.0	16.0	17.4	9.3	22.7	13.0	30.0	27.3	13.0	n/a
Exit (coronal view) (mm)	34.9	20.3	29.0	20.7	25.0	9.3	9.7	38.0	30.8	28.2	12.6	16.7
Ellipsoid area of exit (EA) (mm^2^)	1217.1	494.3	1002.3	520.3	683.4	135.9	345.9	776.0	1451.6	1209.4	257.3	n/a

**Table 3:** ANOVA results

Measurement	ANOVA effects (*F*-statistic, *P* value)		Data subsets found (Tukey’s HSD)	
	Clothing state	Viewing plane	Group 1	Group 2
NL	*F*_2, 18_ = 1.24, *p* = NS	*F*_1, 18_ = 0.07, *p* = NS	No subgroups identified	
D1	*F*_2, 18_ = 0.04, *p* = NS	*F*_1, 18_ = 0.40, *p* = NS	No subgroups identified	
H1	*F*_2, 18_ = 2.38, *p* = NS	*F*_1, 18_ = 1.20, *p* = NS	No subgroups identified	
E1	*F*_2, 18_ = 1.30, *p* = NS	*F*_1, 18_ = 0.06, *p* = NS	No subgroups identified	
EA	*F*_2, 8_ = 1.22, *p* = NS	N/A	No subgroups identified	

Damage measurements appear comparable across clothing states overall. NL appeared shorter where C_max_ was used, though this was not significantly different from the other clothing states, probably due to the large coefficient of variation seen. E1 measurements were generally quite large when considering projectile size, though this may be expected where projectiles were yawing prior to striking the target, thus presenting a greater cross-sectional surface area upon limb contact.

Measurements appeared comparable between viewing planes where no significance was found, suggesting wounding patterns were of a relatively uniform shape.

No significant differences were found between clothing states for each series of measurements taken.

## Discussion

Whilst previous work has demonstrated the significant effect of clothing with projectiles striking an extremity wound model [9, 10], it is clear from the current experiments how important a factor projectile yaw is with regard to the resulting wounding pattern.

In contrast to these previous studies, the presence of clothing did not appear to further influence the severity of wounding seen from the damage inflicted upon the model with projectiles already yawing prior to striking their targets.

From a clinical perspective, the smaller and narrower the wound channel, and the less evidence of significant cavitation found, then the less invasive the level of surgical management is required [5, 35, 36]. These results clearly demonstrate wounding patterns which are still substantial and as such would require relatively invasive surgical management compared with more simple through and through soft tissue wounds [9, 10, 37, 38]. The size of temporary cavity formation relative to the yaw of the projectile, though not measured within this study, is clearly increased proportionally to the contact surface area of the projectile with tissues and as such the damage recorded is a reflection of this [9–11, 14, 18, 19]. The use of the 5.45 mm projectile has previously been demonstrated to yaw early within target penetration and despite no evidence of external deformation or fragmentation, it has been found to have internal deformation of the lead tip found above the steel core [17, 19].

The findings from this paper, coupled with other recent studies [9, 10], provide a more realistic expectation of injury patterns that may be expected on the battlefield, where typical engagements with the enemy will be of varied distances, and therefore varied projectile velocity and symmetry.

### Limitations

There were several limitations to consider. The main limitation was the control of yaw. Use of a larger barrel to fire projectiles from ensures an increased precession and nutation as the projectile exits the barrel; however, measuring and reproducing the accuracy of yaw in degrees was not achieved within this experiment.

Clothing was limited to being representative of that worn by UK troops on current operations only; however, this is building into an increasing amount of data being gathered within this field for future comparison [9, 10]. This could be useful to look at other nation’s military clothing or civil service agency clothing such as police, when examining GSW patterns in future studies.

Ammunition was limited to one type. It would be beneficial to test multiple types pertinent to the threats expected by modern troops in combat.

## Conclusion

Clothing state does not influence damage within an extremity GSW model where projectiles yaw before striking the target. Projectile yaw is therefore likely a key variable with regard to causation of damage within this extremity wound model.

## Footnotes

1 5.45 × 39 mm; mild steel core, 53 grain full metal jacket, Lot number 539-04, made in Russia, 2004; with a core composition of steel; a core tip composition of lead was found, and for the jacket, the composition found to be steel with internal and external copper wash; mean hardness was 814.9 Hv for the core, 3.6 Hv for the core tip and 188.8 Hv for the jacket [9].

2 C_min_ mean thickness = 0.43 mm; mean mass per unit area = 191.14 g/m^2^ [9]

3 C_max_ mean thickness = 32.26 mm; mean mass per unit area = 7735.17 g/m^2^ [9]

4 Phantom V12 video camera (frames per second = 28,000, shutter speed = 4 μs, resolution = 512 × 384)

5 Phantom V1212 video camera (frames per second = 37,000, shutter speed = 5 μs, resolution = 512 × 384)
